# Sr_2_Fe_1.575_Mo_0.5_O_6-*δ*_ Promotes the Conversion of Methane to Ethylene and Ethane

**DOI:** 10.3390/membranes12090822

**Published:** 2022-08-23

**Authors:** Shiqi Song, Lingting Ye, Kui Xie

**Affiliations:** 1 Key Laboratory of Design and Assembly of Functional Nanostructures, Fujian Institute of Research on the Structure of Matter, Chinese Academy of Sciences, Fuzhou 350002, China; 2 University of Chinese Academy of Sciences, Beijing 100049, China; 3 Key Laboratory of Optoelectronic Materials Chemistry and Physics, Fujian Institute of Research on the Structure of Matter, Chinese Academy of Sciences, Fuzhou 350002, China; 4 Fujian Science & Technology Innovation Laboratory for Optoelectronic Information of China, Fuzhou 350108, China; 5 Advanced Energy Science and Technology Guangdong Laboratory, 29 Sanxin North Road, Huizhou 116023, China

**Keywords:** solid oxide electrolysis cell, metal–oxide interface, oxidative coupling of methane, ethane and ethylene

## Abstract

Oxidative coupling of methane can produce various valuable products, such as ethane and ethylene, and solid oxide electrolysis cells (SOECs) can electrolyze CH_4_ to produce C_2_H_4_ and C_2_H_6_. In this work, Sr_2_Fe_1.575_Mo_0.5_O_6-*δ*_ electrode materials were prepared by impregnation and in situ precipitation, and Sr_2_Fe_1.5_Mo_0.5_O_6-*δ*_ was taken as a reference to study the role of metal–oxide interfaces in the catalytic process. When the Fe/Sr_2_Fe_1.575_Mo_0.5_O_6-*δ*_ interface is well constructed, the selectivity for C_2_ can reach 78.18% at 850 °C with a potential of 1.2 V, and the conversion rate of CH_4_ is 11.61%. These results further prove that a well-constructed metal–oxide interface significantly improves the catalytic activity and facilitates the reaction.

## 1. Introduction

Transformation of natural gas (methane) into ethane, ethylene, propylene, benzene, methanol and other value-added chemicals in an economic and environmentally-sustainable way is still a tough challenge in chemistry [1,2,3]. CH_4_ is fairly stable since it contains strong C–H bonds (first bond ionization energy: −439.3 kJ·moL^−1^) that are difficult to activate under normal conditions [4]. C_2_H_4_ and C_2_H_6_ (C_2_) are important raw materials in the chemical industry, and there is a large demand gap for C_2_. CH_4_ is more economical than C_2_H_4_ and C_2_H_6_, so it is worth converting CH_4_ to obtain C_2_ [5,6]. At present, C_2_ is produced by steam pyrolysis of CH_4_ and naphtha at high temperatures through a multistage process. However, many impurities appear, and considerable energy is consumed in this process. Moreover, large amounts of CO_2_ are generated, which is inconsistent with the current concept of carbon neutrality and does not meet the requirements of sustainable development.

Therefore, we attempted to utilize oxidative or nonoxidative coupling of CH_4_ for direct conversion of CH_4_ to C_2_, which could eliminate complicated operational steps and reduce costs. In nonoxidative dehydrogenation, C_2_ synthesis requires breaking of the C–H bonds of CH_4_, which requires high energy and production cost. It also results in carbon deposition, which reduces the selectivity for C_2_ in nonoxidative dehydrogenation. Recent studies have shown that the nonoxidative dehydrogenation temperature of CH_4_ is approximately 1090 °C, and the efficiency for conversion of CH_4_ and the selectivity for C_2_ are both approximately 40% [4]. The temperature for oxidative coupling of methane is lower (approximately 800 °C), and the energy consumption decreases greatly. However, it is worth noting that CH_4_ is excessively oxidized to produce CO_2_ and H_2_O. The key is to develop efficient catalysts for oxidative coupling of methane and avoid excessive oxidation. To date, the maximum yield of C_2_ obtained from oxidative coupling of methane is only approximately 26%, and long-term efforts are still needed to achieve satisfactory results.

To advance industrialization of methane oxidative coupling, researchers are seeking suitable reaction systems and catalysts [1,7,8,9,10,11]. The solid oxide electrolytic cell (SOEC) operates at temperatures between 800 and 900 °C, a range which is similar to the temperature required for oxidative coupling of methane [12,13,14,15,16]. We expect research on oxidative coupling of methane with SOECs to provide new insights. In the electrochemical process, the catalytic reaction of methane can be regulated by oxygen ion transmission and activated by the synergistic effects of electrochemical oxidation and nanostructure catalysis at the electrode. Combinations of metal nanoparticles and oxides have been proven to be an effective strategy for producing SOECs to promote catalytic conversion of CH_4_ to C_2_H_4_. Among them, formation of metal–oxide interfaces between iron nanoparticles and oxides promotes efficient catalytic oxidation coupling of methane [17,18]. Metal–oxide interfaces are usually prepared by the impregnation method. However, the nanoparticles formed by the impregnation method tend to agglomerate, leading to degraded performance [19,20]. Another feasible method is to prepare the raw material by doping with an excess of metal elements and reducing the metal at a certain temperature to grow nanoparticles and form metal–oxide interfaces. Compared with the impregnation method, the samples formed by this method have a unique interfacial structure with strong metal–oxide interactions. The strong interfacial interaction also favours the transfer of oxygen ions, which can activate and oxidize CH_4_ to facilitate the reaction [5].

Electrode materials with double-layer perovskite structure, represented by Sr_2_Fe_1.5_Mo_0.5_O_6-*δ*_ (SFMO) materials, have attracted extensive attention in the research of SOECs [21,22]. SFMO is often used as an electrode material for SOECs since it has a double layer perovskite structure and exhibits high conductivity and good redox stability in both oxidized and reduced states [23,24,25]. It is thought that SFMO exhibits good ion and electron transport properties because the electronic structures of Fe and Mo in SFMO and the strong hybridization of Fe/Mo lead to easy formation of oxygen vacancies and electronic defects.

In this paper, Sr_2_Fe_1.575_Mo_0.5_O_6-*δ*_ electrode materials are prepared by impregnation and in situ precipitation. A sample synthesized by the in-situ precipitation method is denoted 0.075Fe(S)-SFMO, and the other obtained by the impregnation method is denoted 0.075Fe(I)-SFMO. To further evaluate the catalytic performance, the prepared Sr_2_Fe_1.5_Mo_0.5_O_6-*δ*_ was taken as a reference. In the previous study by Xie et al. [5], various samples were prepared by in situ precipitation for experiments. However, in this study, the same samples were prepared by the in-situ precipitation method and the dipping method, and the results were used to judge whether the samples prepared by different methods had the same effect. Upon treating SFMO with reducing conditions, excessive amounts of Fe were dissolved from the B-site; therefore, nanocrystalline iron particles were grown to form metal–oxide interfaces and enhance the oxidative coupling of methane. We further used a series of SFMO materials to construct symmetric SOECs with a La_0.9_Sr_0.1_Ga_0.8_Mg_0.2_O_3_ (LSGM) electrolyte prepared via solid phase synthesis. The SOECs induced conversion of CH_4_ to C_2_ at 850 °C. The SOEC equipped with a symmetric Sr_2_Fe_1.575_Mo_0.5_O_6-*δ*_ electrode material exhibited good oxidative coupling of methane with 11.61% conversion of CH_4_ and 78.18% selectivity for C_2_ at 850 °C with an applied potential of 1.2 V. Engineering of the constructed metal–oxide interface efficiently converts CH_4_ into C_2_ and provides important value for design and application of SOECs.

## 2. Methods

### 2.1. Materials Syntheses

The perovskite SFMO and 0.075Fe(S)-SFMO powders were prepared by the glycine-citric acid combustion method with strontium nitrate, ferric nitrate and ammonium molybdate used as precursors. Then, they were kept at 1200 °C for 5 h in air. It should be noted that the raw material ratios for 0.075Fe(S)-SFMO and SFMO were different. The 0.075Fe(I)-SFMO powder was obtained from the above SFMO powder. SFMO powder was quantitatively added to a ferric nitrate solution according to the stoichiometric ratio of elemental iron, and then the impregnated 0.075Fe(I)-SFMO powder was obtained after stirring and drying evenly.

We also used glycine-citric combustion to synthesize Ce_0.8_Sm_0.2_O_2-*δ*_ (SDC) powder. La_0.9_Sr_0.1_Ga_0.8_Mg_0.2_O_3_ (LSGM) powder was obtained via a solid-state reaction and heated at 1000 °C for 6 h. Powdered sample (0.8 g) was pressed into a sheet with a diameter of 20 mm in a mould under a pressure of 8 MPa. The pressed electrolyte sheet was calcined at 1450 °C for 11 h to obtain the ceramic electrolyte.

### 2.2. Cell Fabrication

Appropriate amounts of SFMO and SDC powder (65:35 ratio) were mixed into an electrode slurry consisting of terpineol, tapioca starch and ethyl cellulose. Then, the samples were evenly coated on both sides of the LSGM surface and heated for 3 h at 1100 °C to form cell pellets. We used silver paste to make a collector layer, and a silver wire was connected to the silver paste on each side of the cell pellet, which was then treated in air at 550 °C for 30 min. In this way, SOECs assembled with a symmetric SFMO-SDC electrode were obtained. The symmetrical 0.075F(S)-SFMO-SDC and 0.075Fe(I)-SFMO-SDC electrode materials were treated with the same procedures to obtain SOECs. Before methane oxidative coupling was performed, a small amount of asbestos was added, and then 5% H_2_/Ar was supplied to the anode at 850 °C to activate the anode material. Electrochemical data for conversion of methane to C_2_ were collected at 850 °C with an electrochemical workstation (Zahner IM6, Zahner Electric, Kronah, Germany). The C_2_ in the output gas was analysed using gas chromatography (GC-2014, SHIMADZU, Kyoto, Japan).

### 2.3. Experimental Characterizations

X-ray diffraction (XRD, Miniflex600, Rigaku, Tokyo, Japan) was used to verify formation of the SFMO material phase. The elemental valence states in the reduced and oxidized samples were analysed by X-ray photoelectron spectroscopy (XPS, ESCALAB 250Xi, Thermo Fisher, Waltham, MA, USA). The microstructures of the electrodes were observed by scanning electron microscopy (SEM, SU-8010, Tokyo, Japan) and high-resolution transmission electron microscopy (HRTEM, Tecnai F20, Hillsboro, OR, USA).

## 3. Results and Discussion

**Figure 1:**
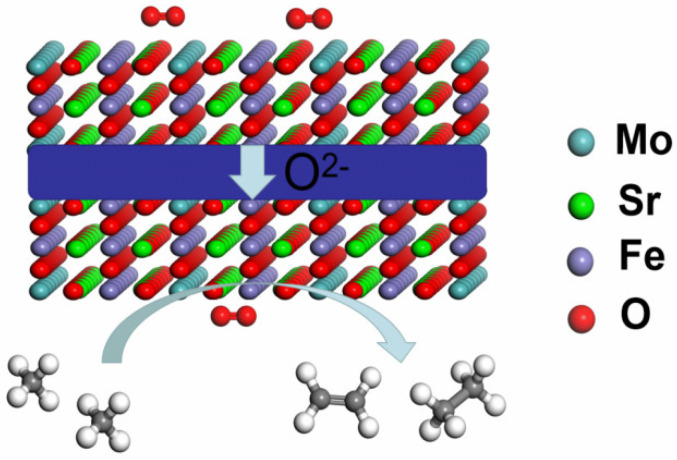
Electrochemical oxidation of CH_4_ to chemicals. Anode electrode: 4CH_4_ + 3O^2−^ → C_2_H_4_ + C_2_H_6_ + 3H_2_O + 6e^−^. Cathode electrode: O_2_ + 4e^−^→2O^2−^.

As shown in Figure 1, the cathode reaction is O_2_ + 4e^−^ → 2O^2−^, and oxygen ions are transported through the electrolyte membrane to the anode in the oxygen conducting SOEC [26,27,28]. When CH_4_ is supplied to the anode, the anodic reaction is 4CH_4_ + 3O^2−^ → C_2_H_4_ + C_2_H_6_ + 3H_2_O + 6e^−^. However, the C_2_H_4_ and C_2_H_6_ products are also easily oxidized to CO_2_. 

**Figure 2:**
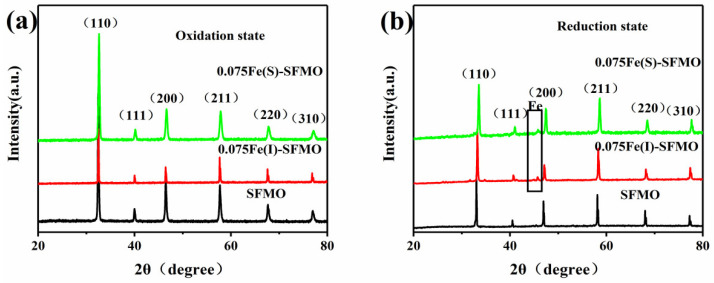
XRD characterization of SFMO samples. XRD patterns of samples after pretreatment in (**a**) air and (**b**) 5% H_2_/Ar.

The XRD patterns for SFMO, 0.075Fe(S)-SFMO and 0.075Fe(I)-SFMO in the oxidized and reduced states are shown in Figure 2, which revealed that they all adopted clear cubic perovskite structures. As shown in Figure 2b, a new peak appeared at 44.76°, which corresponded to the (110) plane of metallic iron, for 0.075Fe(S)-SFMO and 0.075Fe(I)-SFMO reduced at 850 °C with 5% H_2_/Ar. While the iron nanoparticles were successfully exsolved from the lattice, the perovskite structure remained unchanged, which further implied that although the excess iron in the B site was precipitated as metallic iron, the oxide still had stable oxidation-reduction properties.

**Figure 3:**
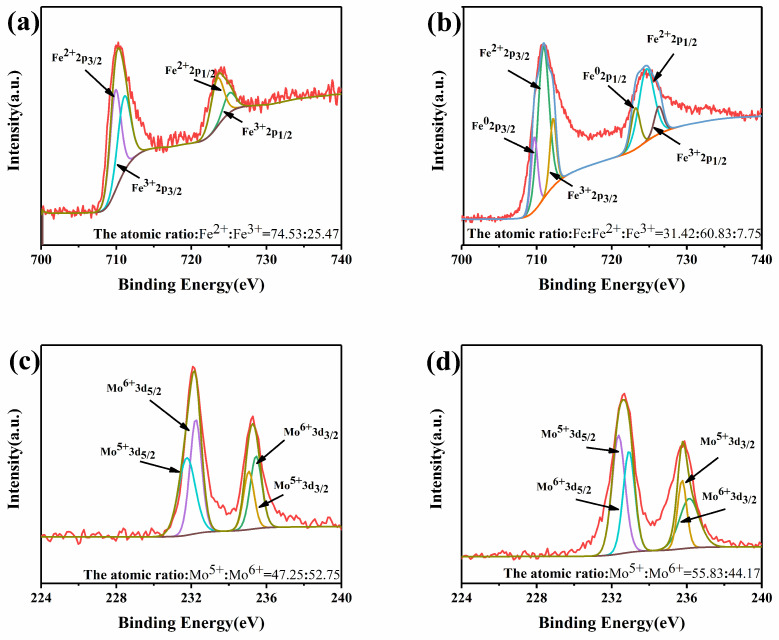
The chemical state of the elements. Fe 2p (**a**) and Mo 3d (**c**) XPS of oxidized 0.075Fe(S)-SFMO; Fe 2p (**b**) and Mo 3d (**d**) XPS of reduced 0.075Fe(S)-SFMO.

Figure 3 shows the valence states of Fe/Mo in 0.075Fe(S)-SFMO for the oxidized and reduced states. Fe existed as Fe^3+^ and Fe^2+^ for the oxidized state of 0.075Fe(S)-SFMO. In contrast, reduced 0.075Fe(S)-SFMO exhibited metallic iron at B sites in the lattice. Part of the Fe^3+^ was reduced to a low energy state in the lattice, but the perovskite structure remained intact. Fe^2+^ was reduced to metallic Fe after pretreatment in a H_2_/Ar atmosphere, consistent with the XRD results. The valence ratios in the oxidized state and reduced state were different, which resulted in different valence states for both Fe and Mo in Figure 3. In theory, changes in valence states can lead to lattice distortions, which are beneficial for transport of ions and electrons. 

**Figure 4:**
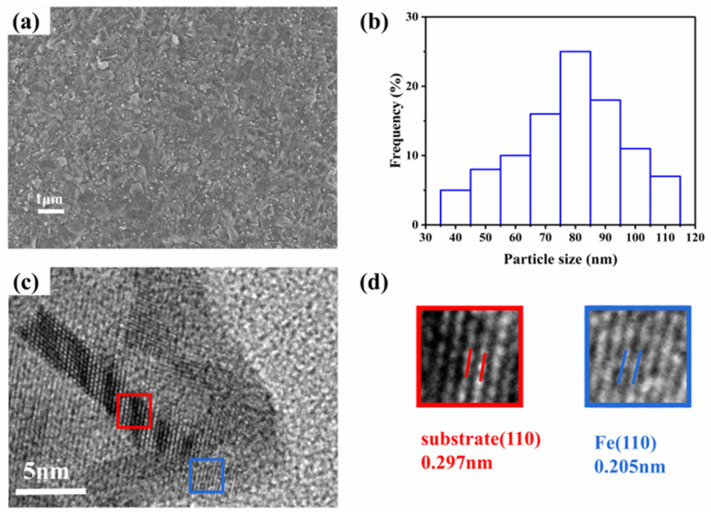
Morphological characterization of the sample. (**a**) SEM image, (**b**) plot of frequency vs. particle sizes, (**c**) TEM image, and (**d**) high−resolution TEM representing the lattice distance (lines of red and blue).

Figure 4 shows SEM and HRTEM images for 0.075Fe(S)-SFMO after 20 h of reduction with 5% H_2_/Ar at 850 °C. Figure 4a shows that the iron nanoparticles were evenly distributed on the surface of the sample, and they were firmly bonded to the 0.075Fe(S)-SFMO surfaces with an average particle diameter of approximately 90 nm. The TEM image of the reduced 0.075Fe(S)-SFMO sample (Figure 4c) suggested that the Fe nanoparticles were anchored on the surfaces of the 0.075Fe(S)-SFMO substrate. The plane spacing of 0.278 nm referred to the (110) plane. HRTEM images further showed that the lattice spacing of the Fe particles was 0.205 nm, corresponding to the (110) plane, which was consistent with the XRD results.

**Figure 5:**
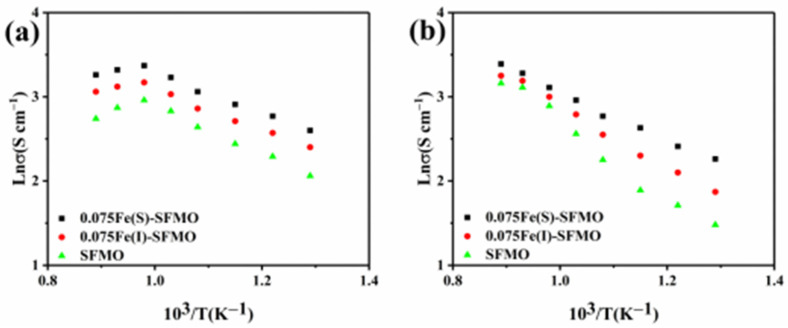
Conductivity of the samples. Total conductivity of SFMO materials in (**a**) air and (**b**) 5% H_2_/Ar.

The relationship between conductivity of the samples and the temperature before and after reduction in a H_2_/Ar atmosphere is shown in Figure 5. The conductivity of the SFMO material in the oxidized state gradually increased to a maximum value and then decreased with increasing temperature, as shown in Figure 5a. The maximum conductivity of the 0.075Fe(S)-SFMO sample was 28.78 S cm^−1^ when the temperature reached 750 °C. In addition, conductivity of the sample in the reduced state also increased with increasing temperature and was 29.66 S cm^−1^ for 0.075Fe(S)-SFMO heated to 850 °C (Figure 5b). This showed that 0.075Fe(S)-SFMO had a higher conductivity than the other materials, which could lead to higher activity. The reason for the excellent electrical conductivity of the SFMO material is that metal Fe NPs were exsolved by reduction in a 5% H_2_/Ar atmosphere and fixed on the surface of the electrode material, thus improving the electrical conductivity of the sample.

**Figure 6:**
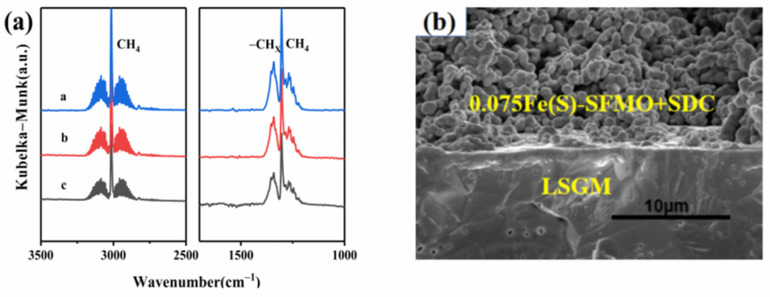
Methane adsorption and the cell morphology. (**a**) FT−IR spectroscopy of CH_4_ for electrode powders (a: 0.075Fe(S)-SFMO, b: 0.075Fe(I)-SFMO, c: SFMO); (**b**) cross−sectional SEM results for 0.075Fe(S)-SFMO-SDC before test.

Figure 6a shows the CH_4_ adsorption capacities of the reduced samples after pretreatment in 5% H_2_/Ar. The absorption peak for 0.075Fe(S)-SFMO was stronger than those of the other samples, indicating that the presence of the Fe/0.075Fe(S)-SFMO interface improved CH_4_ adsorption. Figure 6b displays an SEM image of 0.075Fe(S)-SFMO on LSGM before the test; the top region is a porous 0.075Fe(S)-SFMO-SDC electrode, indicating that the LSGM electrolyte can adhere to the porous structure well. 

**Figure 7:**
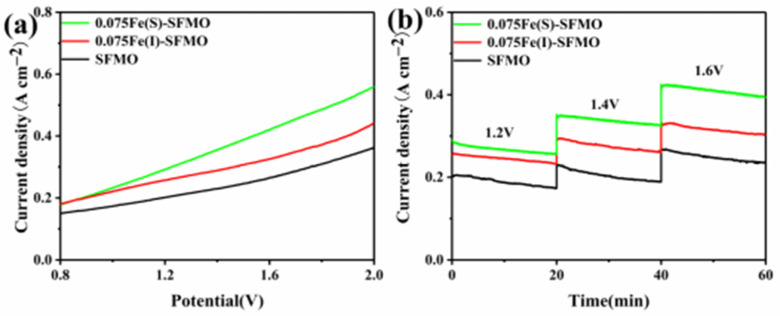
Current density of the samples. (**a**) I−V curves, (**b**) short−term performances of SFMO materials.

Figure 7a shows current density curves obtained for different samples at applied potentials of 0.8–2.0 V. The current density of 0.075Fe(S)-SFMO reached ~560 mA cm^−2^ at 2.0 V and 850 °C, and these values were approximately 70% and 30% higher than those for SFMO and 0.075Fe(I)-SFMO, respectively. Figure 7b shows short-term current curves (20 min) seen with applied potentials of 1.2, 1.4 and 1.6 V, respectively. The current densities of these samples decreased slightly with time and remained basically stable overall. When the potential was 1.6 V, the current density of 0.075Fe(S)-SFMO reached ~420 mA cm^−2^, which was ~1.5 times and ~1.2 times more than those of SFMO and 0.075Fe(I)-SFMO, respectively. The increase in current density was due to the active metal–oxide interface formed by exsolution of iron nanoparticles on the sample surface. In addition, it was the iron nanoparticles precipitated from the perovskite that provided close contact at the interface between the perovskite scaffold and metal iron particles, thus improving the thermal stability at high temperature.

**Figure 8:**
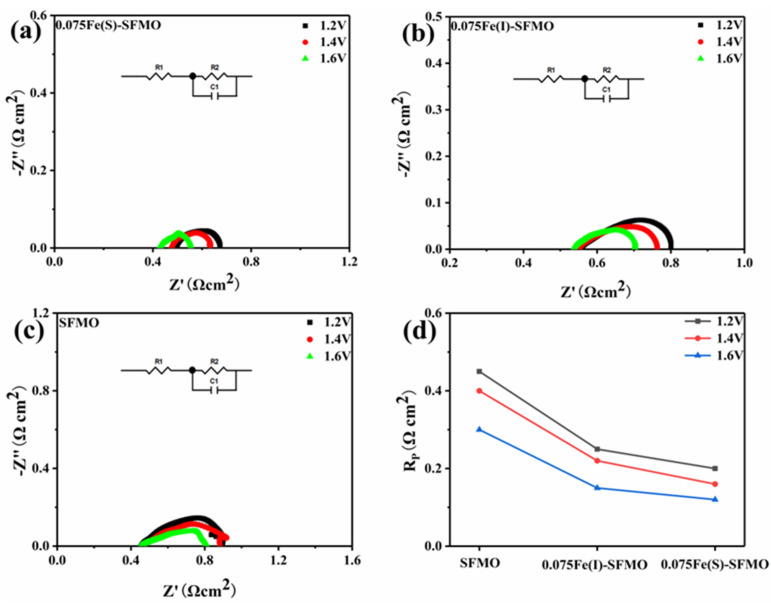
Electrochemical performance of SFMO materials. AC impedance spectra based on (**a**) 0.075Fe(S)-SFMO, (**b**) 0.075Fe(I)-SFMO and (**c**) SFMO. (**d**) The electrode polarizations with different anodes at 1.2–1.6 V at 850 °C.

Figure 8a–c shows AC impedance spectra for the different electrodes at different potentials. The polarization resistance *R_p_* decreased for all samples when the applied potential was increased from 1.2 V to 1.6 V, which means that a higher potential is helpful in activating the electrode and improving its activity. Figure 8d shows a summary of *R_p_* values obtained with the different electrode materials at potentials ranging from 1.2 V to 1.6 V. Obviously, the polarization resistance values of symmetric SOECs assembled with different SFMO materials were roughly similar. However, both 0.075Fe(S)-SFMO and 0.075Fe(I)-SFMO with metal–oxide interfaces exhibited lower *R_p_* values than SFMO without an interface. The 0.075Fe(S)-SFMO material showed a lower Rp than 0.075Fe(I)-SFMO. These results indicated that a well-structured interface activated the electrode and improved the electrolytic activity, resulting in excellent polarization resistance.

**Figure 9:**
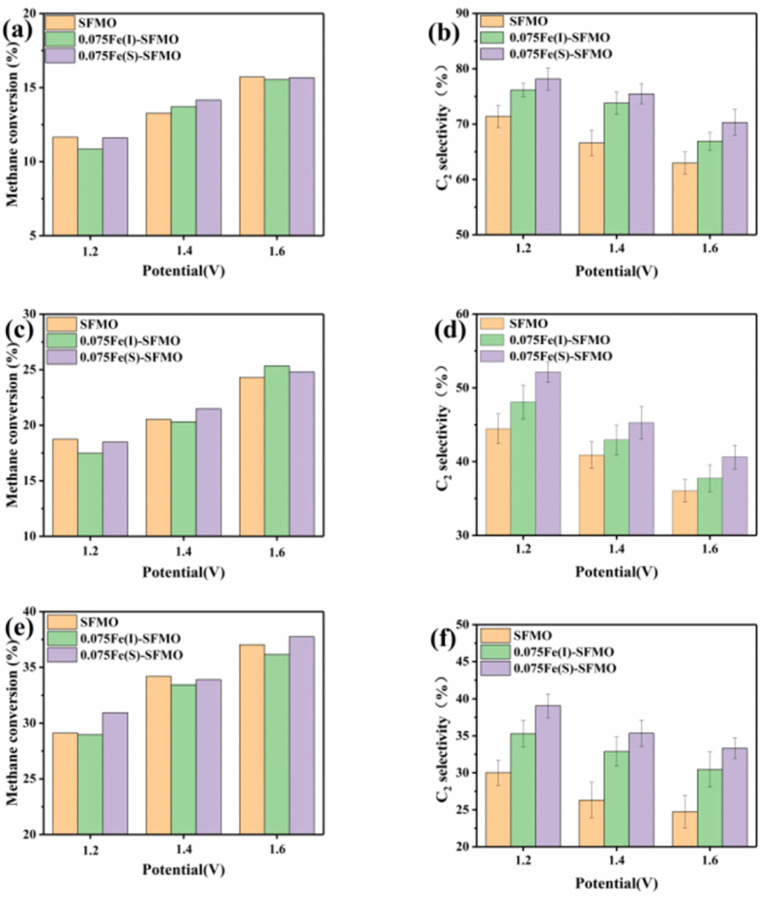
The performance of methane oxidative coupling. CH_4_ conversion (**a**) and C_2_ selectivity (**b**) at the rate of 0.3 L min^−1^; CH_4_ conversion (**c**) and C_2_ selectivity (**d**) at the rate of 0.2 L min^−1^; CH_4_ conversion (**e**) and C_2_ selectivity (**f**) at the rate of 0.1 L min^−1^.

Figure 9 shows the electrochemical oxidation products produced from CH_4_ at 850 °C and with different SOECs. The reactions took place when oxygen ions were pumped from the air at the cathode to the anode. CH_4_ was supplied to the anode at a concentration of 10%, and the product in the anode exhaust stream was analysed with on-line gas chromatography. The methane conversion increased with increasing applied potential from 1.2–1.6 V at a flow rate of 0.3 L/min, and there was little difference in the methane conversion of each component at the same voltage, as shown in Figure 9a. Figure 9b shows the C_2_ selectivity observed at the same flow rate (0.3 L/min), and the C_2_ selectivity of each component decreased with increasing applied voltage. However, the C_2_ selectivity of each component was different at the same voltage, and the C_2_ selectivity decreased in the order 0.075Fe(S)-SFMO, 0.075Fe(I)-SFMO and SFMO, which indicated that 0.075Fe(S)-SFMO exhibited the highest selectivity for conversion of CH_4_ to C_2_. The same conclusion was obtained for different flow rates, as shown in Figure 9c–f. Among them, the highest C_2_ selectivity was that of 0.075Fe(S)-SFMO at 78.18%, and the methane conversion was 11.61% at 850 °C with a flow rate of 0.3 L/min and a potential of 1.2 V. These results indicated that precipitation of iron nanoparticles improved the conversion of CH_4_ to C_2_ under the same operating conditions for different anodes.

## 4. Conclusions

In conclusion, we demonstrated a different catalytic system for efficient and selective conversion of CH_4_ to C_2_ in solid oxide electrolytic cells. When electrochemical oxidation of CH_4_ in the anode was combined with O_2_ electrolysis in the cathode, the oxygen ions were pumped electrochemically to the metal–oxide interface of the anode, which enabled carbon removal and activation of CH_4_. We prepared a series of SFMO electrode materials and successfully constructed metal–oxide interfaces. SFMO electrode materials were used to study oxidative coupling of methane. Through the comparison of SFMO, 0.075Fe(S)-SFMO and 0.075Fe(I)-SFMO, it can be proved that the existence of metal–oxide interfaces can promote the conversion of CH_4_ to C_2_. During further comparison of 0.075Fe(S)-SFMO and 0.075Fe(I)-SFMO, the 0.075Fe(S)-SFMO sample exhibited better performance, and the C_2_ selectivity reached 78.18% with 11.61% methane conversion at 850 °C. This demonstrated that the excellent growth of metal–oxide interfaces can generate interfacial interactions and promote catalytic transformations.

## Data Availability

The data presented in this study are available in this article.
